# Assessing factorial invariance of two-way rating designs using three-way methods

**DOI:** 10.3389/fpsyg.2014.01495

**Published:** 2015-01-08

**Authors:** Pieter M. Kroonenberg

**Affiliations:** Department of Child and Family Studies, Institute of Education and Child Studies, Leiden UniversityLeiden, The Netherlands

**Keywords:** stimulus-response data, Tucker3 model, Tucker2 model, Parafac model, three-mode analysis, rating scales, semantic differentials, Ainsworth strange situation

## Abstract

Assessing the factorial invariance of two-way rating designs such as ratings of concepts on several scales by different groups can be carried out with three-way models such as the Parafac and Tucker models. By their definitions these models are double-metric factorially invariant. The differences between these models lie in their handling of the links between the concept and scale spaces. These links may consist of unrestricted linking (Tucker2 model), invariant component covariances but variable variances per group and per component (Parafac model), zero covariances and variances different per group but not per component (Replicated Tucker3 model) and strict invariance (Component analysis on the average matrix). This hierarchy of invariant models, and the procedures by which to evaluate the models against each other, is illustrated in some detail with an international data set from attachment theory.

## 1. Introduction

Two-way rating designs may consist of, for instance, ratings of concepts on several rating scales. In this paper we tackle the problem of the invariance of the factorial structure of data arising from such designs when the data have been collected from several groups. In particular we will show that three-mode component models are ideally suited to assess factorial invariance for such designs. We will specify a hierarchy of models with increasing restrictions on the parameters resulting in more and more invariant factorial structures across groups.

Because in this paper we are dealing with component models we will use the term “components” rather than “factors,” unlessfactors are explicitly indicated. However, to stay within the standard terminology we will use the term *factorial invariance*, rather than *subspace invariance* or *component invariance*. A detailed treatment of the differences between factor analysis and component analysis for two-way data can for instance be found in Widaman (2007).

### 1.1. Factorial invariance in tests

Most of the research on factorial invariance assumes that an investigator wants to evaluate whether a test with a particular dimensional structure operates in the same way for different groups, so that the test, or the factors underlying it, can be used for all kinds of groups; a detailed technical exposition of measurement invariance, factorial invariance and their relationship can be found in Meredith (1993). Factorial invariance is typically of interest, for instance, when intelligence tests have been translated into other languages and researchers want to establish whether the translated tests function in the same manner as the original. Alternatively, a researcher may want to know whether a test has the same structure for different groups, say both for regular and for clinical samples.

In a literature survey Vandenberg and Lance (2000, pp. 12–13) synthesized common practices in a list of sequential tests to assess the extent of factorial invariance. The steps in their hierarchy of hypotheses are listed below, but we have listed their first step as the final one, because it is the most restrictive of all invariance schemes, i.e., there is no intergroup variability. Here we present a compact version of their descriptions. Finally, we have added a new first step: Lack of factorial invariance. We need this step later on as a reference point or baseline for our analyses. Note that each next step introduces *additional restrictions* on the parameters of the models.

1. *Lack of invariance*: All groups have different factor patterns.
2. *Configural invariance*: Invariant patterns of factor loadings across groups.
3. *Metric invariance* : Invariant values of factor loadings for like items across groups.
∗a *Scalar invariance*: Invariant intercepts of like items regressions on the factor.
∗b *Unique variances invariance*: Invariant unique variances of like items across groups.
c *Invariant factor variances*: Invariant factor variances across groups.
4. *Invariant factor covariance matrices*: Invariant factor covariance matrices across groups.
∗d *Invariant factor means*: Invariant factor means across groups.
5. *Strict invariance*: Invariant factor means and covariance matrices across groups.

The hierarchy is primarily based on investigations using factor analysis within the context of structural equation modeling with and without estimation of the factor means. This means that it contains concepts and parameters characteristic of such models, such as unique variances, factor means and intercepts of regressions of items on factors. In this paper such concepts do not play a role, because our proposals are based on component analysis. In the sequel, the starred steps are therefore excluded for the following reasons: (^*^a, ^*^d) all scales will be centered across concepts for each group (see below), so that means and factor means do not enter into the models; (^*^b) the concept of unique variances does not play a role in component analysis. Note that when referring to Step 5, “Strict invariance,” we will assume only that the covariance matrices are equal across groups, again because the means have already been removed by centering.

The major analytical techniques for establishing the increasingly stricter types of invariance have primarily been structural equation modeling and item response theory as is evident in this special issue. In the hierarchy of hypotheses about factorial invariance it is implied that the models are nested, so that they can be evaluated, or in the context of structural equation models, tested against each other. This means that an a priori choice has to be made about the factor model itself: How many factors and which items are to be regressed on which factors. Therefore, a two-factor model may be invariant in a different way than a three-factor model for the same data. In this paper we will concentrate on series of both two-factor and three-factor models, but we will not attempt to make detailed comparisons between the two series.

Regarding the component models in this paper, comparisons between models are primarily based on the error sums of squares in relation to their degrees of freedom. These degrees of freedom are calculated as the number of data points minus the number of parameters to be estimated (*N*_parm_) where the means subtracted during the centering of the data are also counted as parameters. Details and formulas for calculating the degrees of freedom for three-way models can for instance be found in Kroonenberg (2008, Section 8.4, p. 177ff).

### 1.2. Two-way rating designs

In psychology a specific kind of measurement design is commonly used, i.e., a *two-way rating design* in which concepts are judged on scales by a number of judges such as in Osgood's classical semantic differential design (Osgood et al., 1957). Alternative two-way rating designs generate stimulus-response data or situation-scale data. Characteristic for the designs is that a subject has to judge to what extent a particular scale or variable pertains to a particular concept or situation. For instance, in a study by Murakami and Kroonenberg (2003), a student had to judge the characteristics of the 24 preludes of Chopin on a number of scales. As example, the student had to indicate whether a prelude of Chopin (concept) is tempestuous or tranquil (scale). Another example, which will be our guiding explanatory case, is the two-way design in which a person with a multiple personality in each personality was asked to judge on a number of scales to what extent a number of concepts pertained to her personal situation. For instance, to what extent she considered her doctor to be good or bad (Osgood and Luria, 1954). The aim in their study was to see whether each personality (Eve White, Eve Black and Jane; each measured twice) used the scales in the same way to rate the concepts.

Yet another kind of two-way rating data results from a design in which for several situations the mean characteristics of groups rather than of individual subjects are described by means of a number of variables. For our detailed example we analyzed a collection of two-way data sets consisting of episodes by variables obtained from several different countries. The data were collected using the Strange Situation, a procedure within the attachment theory paradigm (Ainsworth et al., 1978) (see Section 3).

A two-way rating design seems comparable to multitrait-multimethod (MTMM) designs where the traits and the methods mostly form a fully-crossed design for the response variables. An important difference with the MTMM design is that the two-way rating design is more like a two-way (concept×scale) analysis-of-variance design with the intensity or strength of the judgment by a personality as the response variable.

### 1.3. Two-way rating designs and three-way data

Two-way rating designs produce three-way data because they consist of three ways, i.e., concepts, scales and groups or individuals. For a more detailed discussion of such *three-way rating data* arising from two-way rating designs see Kroonenberg (2008, Chapter 14). As far as we have been able to trace, there is no or hardly no explicit literature on the topic of factorial invariance for two-way rating designs, and with this paper we aim to fill this gap. In particular, our aim is to look for both a consensus structure about the relations between the concepts and scales (i.e., invariance over groups) and for group differences, i.e., deviations from invariance. Even though we will primarily focus on the situation with a limited number of groups or individuals, also larger numbers can be analyzed. The emphasis in the present paper is an exploratory one, even though the comparative evaluation of different aspects of factorial invariance using fit measures is a central concern. However, the sizes and relevance of these differences have to be evaluated subjectively both by comparing fit/degrees of freedom ratios and by looking at substantive relevance and interpretability. Formal statistical testing is not part of the procedure.

### 1.4. Invariance in two-way rating designs

A problem for the invariance analysis of two-way rating designs is that there are often only a limited amount of judges or groups rather than large samples from a population so that there is no clear stochastic element in the data. The judges or groups need to be treated as another fixed factor in the analysis-of-variance sense, so that we really have a three-way design of concepts × scales × groups or concepts × scales × individuals. Even apart from the extremely small samples, this lack of stochastics in two-way rating designs makes using confirmatory factor analysis for testing invariance within the standard structural equation modeling context virtually impossible. Therefore, we propose to seek recourse to variants of component analysis, but it should be noted that the procedures discussed in this paper can handle large random samples as well.

Factorial invariance for two-way rating designs is cast here in a non-stochastic component framework in which we have separate component spaces for the scales and the concepts. This has a disadvantage because components are generally not in themselves meaningful quantities but only maximum variance directions in the component space. What are invariant are the subspaces spanned by the components, rather than the components themselves. Therefore, we cannot automatically assume that the components themselves have intrinsic meaning like factors in confirmatory common factor analysis.

Only in some very specific models, such as the Parafac models which have unique solutions (see below), the components can validly be said to have intrinsic meaning. This will limit the kinds of invariances we can consider. Thus, generally we will have to discuss the invariance of subspaces across groups rather than the invariance of the components themselves. As already indicated in the introduction rather than refer to *subspace invariance* or *componential invariance*, we will use the standard term *factorial invariance*.

The two central questions in two-way rating designs are (1) how to define factorial invariance and (2) how to evaluate it. In contrast with the standard situation of assessing whether factorial invariance exists for a particular test across groups, in a two-way design one has to deal with the fact that *groups* or *individuals* use the rating scales to judge concepts. A definition of factorial invariance in this case must include three aspects of the data: (1) the component space or structure of the scales; (2) the component space or structure of the concepts and (3) the way the concepts (or the concept components) and the scales (or scale components) are linked for each group. The consideration of three different aspects of factorial invariance makes the situation for two-way rating designs fundamentally different from the standard situation. Both because of the design and the fact that we are dealing with component spaces rather than factors, makes that the Vandenberg and Lance steps have to be reformulated.

#### 1.4.1. Preprocessing

Variances of components in standard component analysis are represented by the eigenvalues. Whether they are actual variances or merely corrected or uncorrected sums of squares depends on the preprocessing, i.e., centering and normalization of the data. Standardization is more or less automatically carried out in regular component analysis but in two-way rating designs there are several options for preprocessing. Each option has different consequences for the data to be assessed for invariance, because it influences which part of the data is analyzed (see e.g., Kroonenberg, 2008, Chapter 6). To avoid such complications we will ignore the influence of preprocessing in this paper, and we will use the terms sums-of-squares and variances indiscriminately.

### 1.5. Invariance hierarchy

When adapting the steps in the invariance hierarchy for two-way rating designs, we will assume from the start that we are attempting to approximate the centered data with lower-rank component spaces for the concepts and for the scales. This is in contrast with confirmatory factor analysis where covariance matrices are approximated.

Given the definition of a component, i.e., a linear combination of the original variables, any component is always present in a data set with the same variables given its coefficients; a property called *perfect congruence*; for a detailed discussion of this property see Ten Berge (1986a,b). What is generally different in different data sets with the same variables is the amount of variance explained by the components in each group. When it is not the full component space that is under consideration but only a limited number of (maximum variance) components, these group component spaces can be spanned by different linear combinations of the variables, so that component spaces of different groups may even be orthogonal to each other. The maximum variance components of one group, may account for very little variability in another group.

#### 1.5.1. Step 1. Lack of invariance

The most extreme form of lack of invariance is that each group has its own low-dimensional subspace. For two-way designs we take as our starting point the separate analyses of the group data without imposing any restrictions on the component subspaces other than considering a limited number of components, the same number for each group. The fitted sum of squares of the groups together, the *combined fit*, is calculated by summing their individual fitted sums of squares.

#### 1.5.2. Step 2. Configural invariance

Because every component returns in each data set with the same variables, i.e., components are always perfectly congruent across groups, configural invariance is not a limiting restriction in component analysis and is automatically true. Thus, it cannot be used as a limiting concept in a hierarchy of models, even though in different groups the same components may account for different amounts of variance and have different correlations.

#### 1.5.3. Step 3. Metric invariance

Of the models used to inspect factorial invariance, metric invariance is part of their definition. Thus, the component spaces (for the concepts and scales) specified in the models are such that the component coefficients are identical across groups. Three models can be used to investigate metric invariance. They have either (3a) an invariant concept component space, (3b) an invariant scale space or (3c) both. Metric invariance can be compared with a total lack of invariance by comparing the metric-invariant model fit with the *combined fit*. In addition, the metric invariant space can be compared with the separate spaces of the groups, for instance via Procrustes techniques (see, for instance, Gower and Dijksterhuis, 2004); see also Section 4.

**Figure 1:**
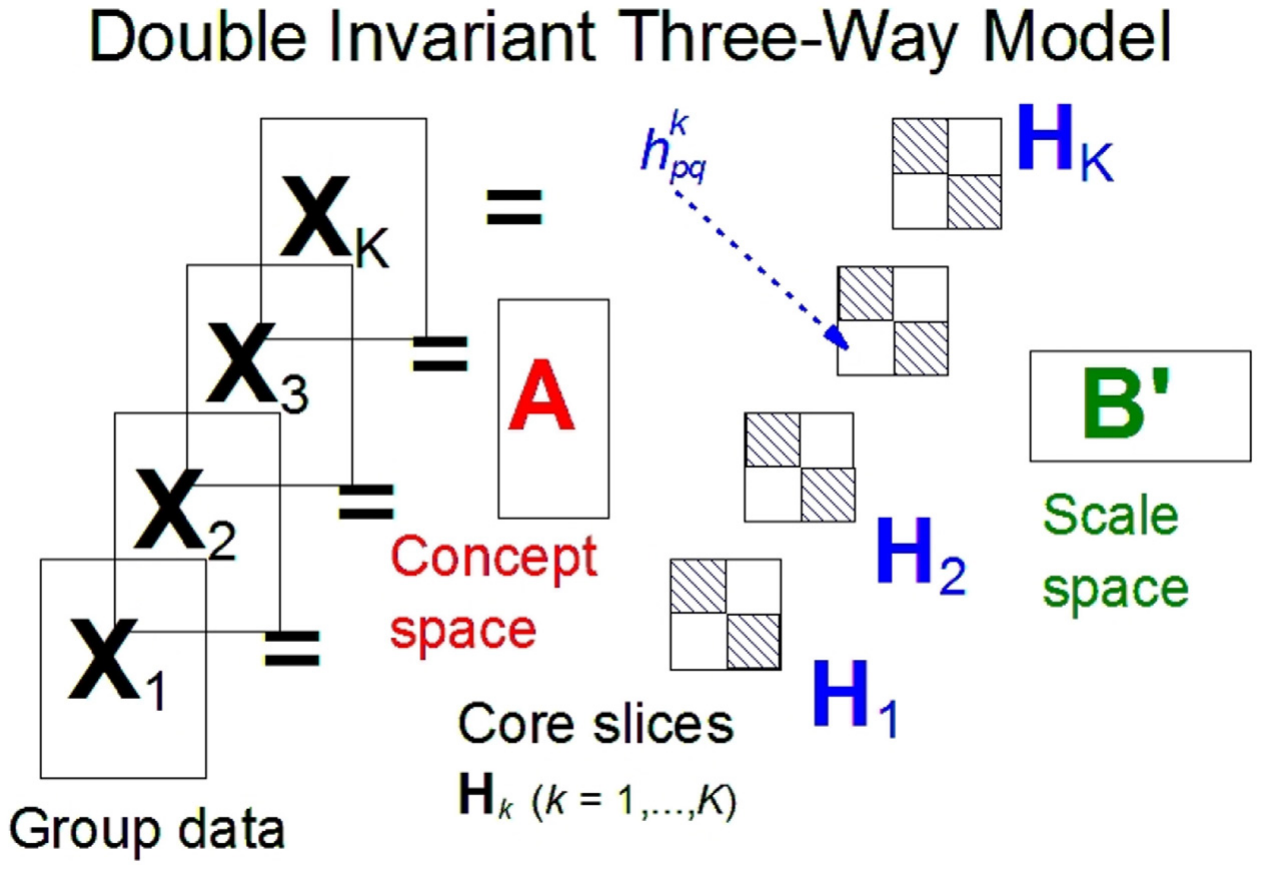
**A general three-mode model for two-way rating designs.** A = metric invariant concept space; B = metric invariant scale space; H_*k*_ = (*h*^*k*^_*pq*_) = core slice for the *k*^*th*^ group; *h*^*k*^_*pq*_ is the link between the *p*th component of A and the *q*th component of B.

For the component models under consideration we will use the terms *links* and *interactions* to indicate the parameters which link the concepts and scales components. The links are contained in a so-called *core array* **H** (see Figure 1). For each group this array contains a slice, **H**_*k*_, with the group's links between the components of the scales and the concepts. If both the concept and the scale space are orthogonal, the sizes of these links are the square roots of variation accounted for by the components. The invariance of the factor covariance matrices across groups translates into the equality of the core slices **H**_*k*_ for *k* = 1, …, *K*.

#### 1.5.4. Step 4. Invariant component covariance matrices or core slices

As no common three-way models have restrictions on the variances without restrictions on the covariances, such models will not be discussed here; see Harshman and Lundy (1984) for detailed considerations about this issue. We will, however, consider (4a) models with invariant covariances (off-diagonal elements of the core slices) for all groups but with different variances (diagonal elements of the core slices). Even more restricted are models in which (4b) the invariant scale and/or concept components are uncorrelated in all groups.

#### 1.5.5. Step 5. (Weighted) strict invariance

The equality of the covariance matrices in Vandenberg and Lance's Step 5 translates into the equality of the centered data matrices of the groups. Such an equality implies equality of random errors which is of course nonsensical. However, a further tightening of the invariance in Step 4 is achieved in Step (5a) by restricting the slices of the core array to be identical, apart from a size coefficient (in the following referred to as a *weight*). Finally, the strictest factorial invariance situation is created in Step (5b) by specifying that also the weights are invariant across groups. In that case the structure of the scales and the concepts, as well as their linkages, are identical in all groups.

### 1.6. Related research

Thus, for the two-way rating design the investigation of invariance is concentrated on the linkages between the invariant components for all groups. The discussion of the hierarchy of increasingly invariant three-mode models in this paper is strongly related to the hierarchy of three-mode models for fully-crossed raw data (Kiers, 1991). In addition, a similar hierarchy can be found in connection with simultaneous component analysis of covariance and correlation matrices (Timmerman and Kiers, 2003). However, in those papers the concept of factorial invariance is not the focus of the investigation nor is the emphasis on two-way rating data.

## 2. Modeling factorial invariance

This section deals with three-way models for analysing data two-way rating designs. These models have as a common characteristic that the scale space and the concept space are invariant for all groups. However, they differ in the nature of the linkages between concept and space components. The models in Step 3a and 3b have *metric invariance* in one mode and all other models are characterized by *double-metric invariance*.

### 2.1. Models for two-way rating designs

Table 1 provides an overview of appropriate models, together with listing the nature of their invariances. To discuss these models in some detail we need some notation. **A** and **B** indicate the *I* × *P* invariant concept space and the *J* × *Q* invariant scale space, with *P* and *Q* the number of components, respectively. A subscript *k* indicates that a particular matrix belongs to the *k*th of *K* groups or levels of the third way; for instance, **X**_*k*_ is the concept × scale data matrix of the *k*th group. **H**_*k*_ = (*h*^*k*^_*ss*_) is the linkage matrix for the concept and the scale components for the *k*th group, **D**_*k*_ is a diagonal matrix of links used in the SVD as well as in the Parafac model. In the next section we will discuss these models in detail and indicate how they embody factorial invariance. As indicated in Table 1 the Tucker2 model in principle allows for different numbers of components for the scales and the concepts, but as it is the only three-way model in Table 1 for which this is the case, we will assume in the following that *S* = *P* = *Q*, i.e., that the numbers of components for the two spaces are the same throughout, so that **A** has size *I* × *S* and **B** has size *J* × *S*.

**Table 1:** **Models for two-way rating designs and their invariance**.

**Model**	**Concepts**	**Scale**	***P* = *Q*?**	**Interaction**	**Abbreviation**
**STEP 1: LACK OF INVARIANCE**					
SVD per group	-	-	yes	no explicit invariance restrictions	SVD_*s*
**STEP 3: METRIC INVARIANCE**					
Tucker1 - concepts invariant	x	-	no	concept space invariant; *single metric invariance*	T1A_*s*
Tucker1 - scales invariant	-	x	no	scale space invariant *single metric invariance*	T1B_*s*
Tucker2	x	x	no	concept and scale spaces invariant; *double-metric invariance*	T2_*ss*
**STEP 4: INVARIANT COMPONENT COVARIANCES**					
Parafac	x	x	yes	+ component covariances invariant; variances free	PF*s*
Parafac - Orthogonal	x	x	yes	+ component covariances invariant; variances free; components orthogonal for one or both ways	PF*s*_Orth
**STEP 5: (WEIGHTED) STRICT INVARIANCE**					
Tucker3 - Free	x	x	yes	metric invariance of orthogonal components variances invariant; group weights unrestricted	T3_*ss*1
Tucker3 - Fixed	x	x	yes	+ group weights fixed and constant	T3_*ss*1Fixed

x = invariant; S-S = not invariant; SVD, Singular Value Decomposition; P = number of concept components; Q = number of scale components; s = 2 or 3 number of components; ss1 = the first two ways have s components, the third way 1 component.

### 2.2. Step 1: singular value decomposition per group

The singular value decomposition (SVD) is the motor of many multivariate techniques. For any **X**_*k*_ it may be written as:

(1) $$X_{k}=A_{k}D_{k}B_{k}^{\prime }+E_{k}={\hat{X}}_{k}+E_{k}\;\;\;\;k=1,\;\cdots ,\;K$$

where for the SVD to have the form in Equation (1), the concept spaces **A**_*k*_ and scale spaces **B**_*k*_ have to have orthogonal components and the linkage matrices **D**_*k*_ have to be diagonal. The **E**_*k*_ contain the errors of approximation. $\hat{X}$_*k*_ = **A**_*k*_ **D**_*k*_ **B**′_*k*_, and **E**_*k*_ = 0 if all components are used. We will refer to the collection of independent analyses for each group as the *separate-analyses model* with abbreviations SVD_2 and SVD_3 for the two- and three-component models, respectively.

Thus, each data matrix **X**_*k*_ has its own decomposition as in Equation (1), and this decomposition is unrelated to that of any of the other data matrices. The total variance of a group *k* is equal to the sum of the squares of the singular values *d*^*k*^_*ss*_ that make up the diagonal of **D**_*k*_ in the full decomposition, i.e., SS(Total)_*k*_ = ∑_*k*_ *d*^*k*^_*ss*_. Adding the SS(Total)_*k*_ of the groups gives the total amount of variance of the groups indicated by SS(Total). In general, we will use only a limited number of components, here either 2 or 3. The components (columns) of **A**_*k*_ and **B**_*k*_ successively account for the largest amount of variance so that, given the dimensionality, the components for the concepts and those for the scales span the subspaces with the highest variance. Thus, we can use this variance accounted for, SS(Fit)_separate_, as an upper bound for the variance accounted for from any other model given the number of components. If the SS(Fit) is the fit for a common model for all *K* groups, then if SS(Fit)_model_ ≃ SS(Fit)_separate_ the component space(s) are invariant. However, if there is a sizeable difference, the invariance restrictions on the common model are in doubt. We may also investigate *group invariance* by comparing the fitted variance of a particular group SS(Fit)_*k*_ with the similar quantity calculated via the parameter estimates from one of the fitted models. Given the number of components, this will provide information on which groups fit well and which groups do not and are thus not invariant with respect to the other groups.

### 2.3. Step 3a and Step 3b: single metric invariance - Tucker1 models

The first step into imposing restrictions on the solutions to investigate possible invariance is to demand that either the concept spaces can be properly represented by a single space (i.e., for all *k* the concept spaces are equal: **A**_*k*_ = **A**), or that for all *k* the scale spaces are equal: **B**_*k*_ = **B** if there are *s* components. This can be investigated with the Tucker1 model, here referred to as Tucker1A (or T1A_*s*) for concept space equality and Tucker1B (T1B_*s*) for scale space equality. Metric invariance exists for the concepts if

(2) $$X_{k}=ADB_{k}^{\prime }+E_{k}\;\;\;\;k=1,\;\cdots ,\;K.$$

Thus, there is a single orthogonal concept space for all *k* and separate scale spaces for each group. Metric invariance exists for the scales if

(3) $$X_{k}=A_{k}\overset{˘}{D}B^{\prime }+E_{k}\;\;\;\;k=1,\;\cdots ,\;K.$$

Thus, there is a single orthogonal scale space for all *k* and separate concept spaces for each group.

To compute the parameters, the three-way array is first converted to a two-way matrix of (Groups × Scales) by Concepts or (Groups × Concepts) by Scales, and these matrices are then subjected to a SVD. Note that the resulting **A**_*k*_ and **B**_*k*_ are no longer orthogonal because they are parts of a single orthogonal matrix of left and right singular vectors, respectively. We may compare the fitted variance of these models SS(Fit)_model_ with the combined results of the separate SVDs, SS(Fit)_separate_, to investigate the metric invariance of either the concept or the scale spaces. However, it seems a bit odd to have an invariant concept space without having an invariant scale space, so we will not include the Tucker1A model further in our deliberations.

### 2.4. Step 3c: double-metric invariance - Tucker2 model

The next step in imposing invariance is to require *double-metric invariance*, i.e., for all *k* and given a number of components *s* both **A**_*k*_ = **A** and **B**_*k*_ = **B**, where both matrices orthogonal. Furthermore, the group linkage matrices **H**_*k*_ are unrestricted and thus in general not diagonal. The model equation for the Tucker2 model (Tucker, 1972), as the model is commonly known (see Kroonenberg, 2008, Section 4.5.2) becomes

(4) $$X_{k}=AH_{k}B^{\prime }+E_{k}\;\;\;\;k=1,\;\cdots ,\;K.$$

In other words, the metric invariance is present on both the concept space and the scale space, and the only differences between the groups can occur in the *K* interaction or linkage matrices, **H**_*k*_. The linkages matrices **H**_*k*_ have sizes *S* × *S*, where *S* is the number of components for both the scale and the concept spaces. An element *h*^*k*^_*pq*_ of **H**_*k*_ represents the link between the *p*th component of the concepts and the *q*the component of the scales for the *k*th group. So apart from their error terms, the variability between the groups lies in the strengths of their links between the concept and scale components or the sizes of the *h*^*k*^_*pq*_.

We can again compare the fitted variance of these models SS(Fit)_model_ with the combined results of the separate SVDs, SS(Fit)_separate_, to investigate the double-metric invariance. Similarly we can make comparisons at group level.

### 2.5. Step 4: double-metric invariance with invariant correlations - Parafac model

By requiring **H**_*k*_ = **C**_*k*_, where the latter are diagonal matrices, and dropping the orthogonality restriction on the component spaces, we get the standard Parafac model with *s* components (PF*s*) which is a double-metric invariant model with as its model equation

(5) $$X_{k}=AC_{k}B^{\prime }+E_{k}\;\;\;\;\;\;\;k=1,\;\cdots ,\;K.$$

The model can also be written by filling the rows of a *K* × *S* matrix $\tilde{C}$ with the diagonals of the **C**_*k*_, i.e., $\tilde{c}$_*ks*_ = *c*^*k*^_*ss*_ *k* = 1, …, *K*. In that case $\tilde{C}$ is considered a component matrix and is normalized like **A** and **B**, i.e., the lengths of the components in all three matrices are equal to one. The sizes of the *S* components are then contained in a diagonal matrix **D** = (*d*_*ss*_). However, for this paper we will stick with the **C**_*k*_.

Harshman (1970) that has shown this model implies that the groups have the same correlations between the components, which is a further imposition of factorial invariance. When at least one of the component matrices is orthogonal the *d*^2^_*ss*_ are the variances of the *S* components.

One can even impose further restrictions on the components and so make the invariance even stricter by reintroducing orthonormality, non-negativity, or unimodality on one or both component matrices (see, e.g., Bro and Sidiropoulos, 1998).

Compared to other three-way models, Parafac models have a special characteristic in that their parameters are uniquely determined under rather mild conditions. This implies that the parameters in Equation (5) cannot by altered, for instance by rotation, without lowering the fit. The consequence is that the model has the *parallel proportional profile* property; (see Cattell and Cattell, 1955; Harshman, 1970; Harshman and Lundy, 1984). The only lack of invariance in these models consists of different strengths of the links between the concepts and scales, i.e., the *c*^*k*^_*ss*_ vary between the groups. From the parallel proportional profile property and the uniqueness of the models it is the components themselves, not only the subspaces they span which are invariant; see Harshman (1970) or Harshman and Lundy (1984).

### 2.6. Step 5: strictly invariant models - Tucker3 models

To study factorial invariance with even more restrictions, we can demand that for each *k c*^*k*^_*ss*_ = *c*^*k*^*d*_*ss*_. In other words the weights for the components are invariant across groups apart from a group weight *c*^*k*^.

(6) $$X_{k}=A(c^{k}D)B^{\prime }+E_{k}=c^{k}(ADB^{\prime })+E_{k}\;\;\;\;\;k=1,\;\cdots ,\;K.$$

This model equals a simplified version of the full Tucker3 model (Tucker, 1966), and has been referred to as the *Replicated PCA* model by Van IJzendoorn and Kroonenberg (1990) and *Weighted PCA* by Krijnen and Kiers (1995). The only variable parts are the weights *c*^*k*^ for the group applicable to both components, and the error terms **E**_*k*_. In other words, all groups have the same concept and scale spaces and the orthogonal components of each way are linked such that each concept component is linked exclusively to a particular scale component. The part between brackets has the form of a SVD valid for all groups. The only differences between the groups are their weights, *c*^*k*^. This is in contrast with the Parafac model where each group has different link weights for the concept and scales component combinations, i.e., the *c*^*k*^_*ss*_ are different for each group *k* and each pair of components *s*.

The ultimate invariant model is that in which we assume that all *c*_*k*_ are all equal with weight *c* = √1/*K*, which is computationally equivalent to first averaging over groups and then carrying out a SVD on the average data matrix **X**, i.e.,

(7) $$X_{k}=\bar{c}(ADB^{\prime })+E_{k}\;\;\;\;\;k=1,\;\cdots ,\;K.$$

Thus, in this case the only variable parts are the error terms and we may speak of *strict invariance*. We could reduce even further the number of parameters by specifying further restrictions on the concept and scale component spaces (see Takane et al., 1995), but this will not be considered here.

### 2.7. Summary evaluating invariance

The conclusion from the above subsections is that one can define a hierarchy of models with an ever increasing number of parameters which are invariant over groups. By comparing the models with each other and with the combined separate analyses, it becomes possible to evaluate which models still provide an adequate fit to the data compared to separate analyses, and hence which type of invariance can be safely adopted. The two leading types of information for this purpose are the overall fitted variance and the fitted variance of each group.

In order to carry out model comparisons the number of parameters estimated for each of the models is determined. The models are compared by constructing a variant of the three-mode scree plot, in which the fitted sum of squares are plotted against the number of parameters estimated (see Section 3.3). Details on how to calculate the number of parameters can be found in Kroonenberg (2008, Section 8.4).

## 3. Example: the strange situation across the world

### 3.1. Research design

Attachment between adults, especially mothers, and infants is a lively research area—(see Cassidy and Shaver, 1999, 2008. Three types of bonds between adults and infants are generally considered: Avoidant attached, Securely-attached, and Resistant/Ambivalent attached, indicated by the letters A, B, and C, respectively. Here we will only look at attachment bonds with mothers, but those with other adults, especially other caregivers, have also been investigated (see, e.g., Sagi et al., 1985). The measurement procedure consists of a series of episodes of approximately 3 min, during each of which the infant is in a standardized room together with the mother (M), the stranger (S), both (MS), or alone (A); the episodes are the following: M1, MS2, S3, M4, A5, S6, M7. The idea is to increase the stress on the infant, especially by introducing the stranger and leaving the child alone, so that the attachment relationship between mother and infant is put to the test. During the episodes, except when the infant is alone (A5), five core variables of an infant's reaction to an adult are measured: Proximity seeking, Contact maintaining, Avoidance, Resistance, and Distance interaction.

#### 3.1.1. Strange situation data set

The data set under consideration consists of 11 samples: US-Belsky (USBel), US-Thompson (USTho), Germany-Berlin (GerBe), Germany-Bielefeld (GerBi), Israel-Kibbutz (IsrKi), Israel-City (IsrCi), Japan-Miyake (JapMi), Japan-Takahashi (JapTa), Netherlands-Younger infants (NLYng), Netherlands-Older infants (NLOld) and Sweden (Swed). The data set was put together by Sagi and Lewkowicz, and in their publication (Sagi and Lewkowicz, 1987) they supply full details of the origins of the different samples. For each of the samples the original investigators independently determined the infants' type of attachment. Earlier analyses can be found in Sagi and Lewkowicz (1987) and Kroonenberg and Van IJzendoorn (1987).

#### 3.1.2. Invariance

The research question for this paper is whether the structure of the scales and that of the episodes, as well as the way these components are linked, are invariant across samples. The more parameters in the models are invariant, the more evidence this presents that the Strange Situation is a valid procedure across countries and researchers. For this example we only examine the average scores of the samples securely attached infants (B). These samples were chosen because each contained a sufficient number of B infants to make the average scores reliable. Thus, the two-way rating design consists of 7 episodes by 5 scales for 11 samples. This three-way data set was subjected to the models described above and their fit measures were compared.

### 3.2. Results: three-way analysis of variance

To acquire an initial perspective on the differences between samples, we carried out a three-way analysis of variance of the Strange Situation data. For this analysis the response variable was considered to be intensity of a reaction, and the Three-Ways were conceived as fixed factors in the ANOVA sense. This view is feasible because the samples are not exchangeable or drawn from a population. Moreover, it is the individual differences between the samples which are the focus of the analysis. Furthermore, the scales all had the same range from 1 to 7, so that averaging across scales is feasible and interpretable.

Table 2 shows that the largest variability is between scales, indicating that the scale scores of the infant-mother dyads are effective in differentiating between behaviors across samples and episodes. On the other hand, the sample variability is comparatively very small (2.2% of the total), indicating that the investigating factorial invariance is a worthwhile exercise. This is confirmed by the size of the episode × scale interaction compared to the interactions involving samples. Finally, the residuals (or the three-way interaction) only take up 7.5% of the total variability.

**Table 2:** **Three-Way analysis of variance (with a single observation per cell)**.

**Source**	**SS**	**% SS(Total)**	***df***	**MS**	***F***
**MAIN EFFECTS**					
Episodes	79.3	16.4%	6	13.2	88.1
Scales	211.4	43.6%	4	52.8	324.0
Samples	10.7	2.2%	10	1.1	6.5
**TWO-WAY INTERACTIONS**					
Episodes × Scales	105.2	21.7%	24	4.4	26.9
Episodes × Samples	9.9	2.0%	60	0.2	1.0
Scales × Samples	29.5	6.1%	40	0.7	4.5
**THREE-WAY INTERACTION**					
Residuals	39.1	8.1%	240	0.2	
**TOTAL**	485.1				

df = degrees of freedom; MS = Mean Sum of squares.

Parallel with standard component analysis, before the three-way analyses the data were centered but not normalized. Normalization was not deemed necessary because all the scales had the same range. Moreover, scales with more variability should be allowed to have more influence on the analysis than scales with little variability.

With respect to centering, the common type of centering for three-way rating scale data (averaging across the concepts) was used, i.e., $\tilde{x}$_*ijk*_ = (*x*_*ijk*_ − *x*_.*jk*_). In other words, the scale means for each sample *k* were removed. In general, centering across samples is undesirable because it will eliminate the consensus configuration of the scales and concepts from the three-way analysis. Thus, due to this type of centering the means of the scales for each of the samples were not included in the invariance analysis, but depending on the purpose of a study, these means can be analyzed for invariance separately.

### 3.3. Results: investigating type of invariance via model fit

Because the procedure outlined for assessing factorial invariance for two-way rating designs is an exploratory one, deciding on the degree of invariance is a substantive and subjective matter, of course based on numerical information. Table 3 provides the information on the series of more and more restricted, and hence more invariant, models. Any additional restriction on the parameters is going to incur a certain amount of additional loss compared to the separate analyses. However, the question is whether the decrease in fit can be acceptable, given that by restricting the number of parameters interpretability is enhanced. It is less useful to compare the two-component models with the three-component models, because they have different starting points, i.e., different separate solutions. Therefore, it seems best to first decide on the number of components one wants to use to model the data, and only after that to investigate the invariance. This is incidentally also the standard practice in structural equation modeling. Of course, one may come to the conclusion that a two-component model is more, or less, invariant than a three-component model and vice versa.

**Table 3:** **Overall sums-of-squares for the Strange Situation data**.

	**Model**	**Abbreviation**	**SS(Fit)**	**SSS(Fit)**	***df***	**N**_**Parms**_
**TWO-COMPONENT SOLUTIONS**						
*Step 1*	SVD per group	(SVD_2)	205.78	0.89	110	275
*Step 3*	Tucker1 - Scales invariant	(T1B_2)	187.94	0.81	227	158
	Tucker2	(T2_22)	177.78	0.76	270	115
*Step 4*	Parafac	(PF2)	173.54	0.74	288	97
	Parafac - Orthogonal scale components	(PF2_Orth)	173.36	0.74	290	95
*Step 5*	Tucker3 + Variable weights	(T3_221)	169.18	0.72	302	83
	Tucker3 + Fixed weights	(T3_221Fixed)	164.77	0.71	312	73
**THREE-COMPONENT SOLUTIONS**						
*Step 1*	SVD per group	(SVD_3)	227.05	0.97	33	352
*Step 3*	Tucker1 - Scales invariant	(T1B_3)	219.53	0.94	154	231
	Tucker2	(T2_33)	206.57	0.88	213	172
*Step 4*	Parafac	(PF3)	200.40	0.86	267	118
	Parafac - Orthogonal scale components	(PF3_Orth)	195.26	0.84	273	112
*Step 5*	Tucker3 + Variable weights	(T3_331)	185.43	0.79	299	86
	Tucker3 + Fixed weights	(T3_331Fixed)	181.23	0.78	309	76

N_Parms_ = Number of parameters (includes 55 removed means due to centering); The Total Sum of Squares of the centered data: SS(Tot) = 233; SSS(Fit) = SS(Fit)/SS(Tot); df = degrees of freedom = number of data points (I × J × K = 385) − N_Parms_.

In Table 3 we see that the most restrictive models are the Tucker3 models with a constant component for the samples (T3-221Fixed and T3-331Fixed), i.e., the strictly invariant models. At the other extreme the individual three-component SVDs are not much use in terms of data reduction, because the model for each sample has only three degrees of freedom, and the rank of the centered data matrices is at most four. From a data-analytic point of view, it is doubtful whether a model with unrestricted three-component solutions for the separate samples is really useful because the three components fit about 97% of the total variability.

**Figure 2:**
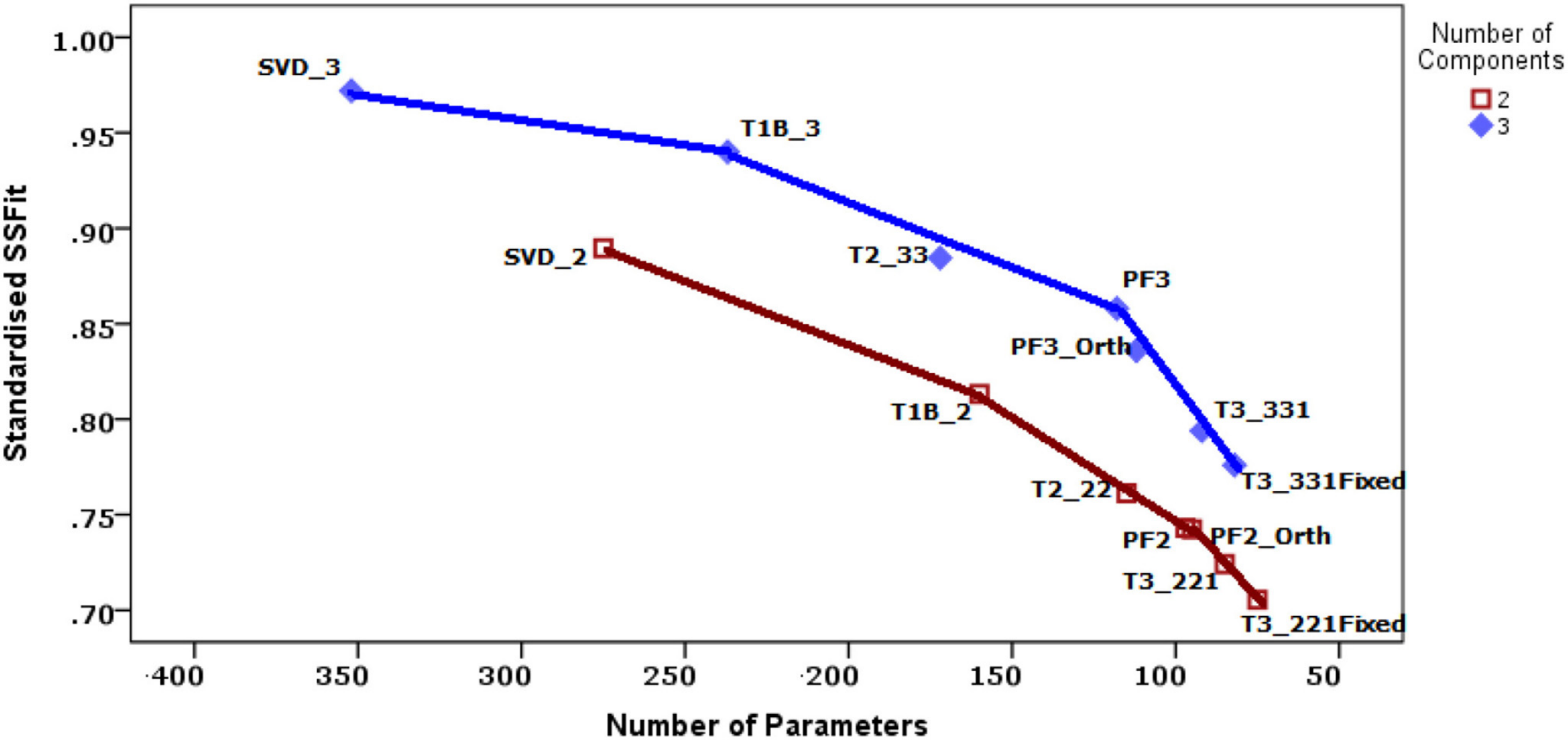
**Model comparisons**. The two-component and three-component models are connected by separate convex hulls. The horizontal axis is reversed because the investigation starts with the individual models. *Legend*: SVD_*s* = separate SVDs with *s* components; T1B_*s* = Tucker1 model with *s* components; T2_*ss* = Tucker2 model with *s* components for way 1 and 2; PF*s* = Parafac model with *s* components; PF*s*_Orth = Parafac model with *s* orthogonal scale components; T3_*ss*1 = Tucker3 model with *s* components for way 1 and 2 and 1 component for way 3; T3_*ss*1Fixed = T3_*ss*1 with a fixed value for way 3.

To decide upon the most appropriate model for these data, and thus on the extent of the invariance, it is useful to construct a variant of the three-mode deviance plot of the fitted sums of squares vs. the number of parameters (Figure 2); (see Kroonenberg, 2008, Section 8.5. The models with two components and those with three have been connected by part of a convex hull. Models on a convex hull are generally preferred to the models inside such a hull because of their more favorable SS(Fit)/*N*_Parms_ ratios. It is preferable to consider only models on or very close to the convex hull; the PF3-Orth model is less attractive because there are models with more favorable ratios (PF3 and T3-331) in the neighborhood. The more horizontal a hull, the more a model on the right is a good alternative for the models to the left on the hull, because the decrease in the number of parameters (i.e., increase in the *df*) does not seriously decrease the fitted sum of squares. In contrast, the steeper the hull turns downward for the next model to the right, the less attractive the model, because there is a large loss in fitted sum-of squares for only a limited decrease in parameters. Note that a smaller number of parameters increases power and potentially simplifies interpretability.

For the Strange Situation data we see in Figure 2 that for the three-component models the convex hull declines slowly at first, and a steeper downturn is observed only for the Tucker3 models, so that the Parafac model with three components seems a good choice. The choice for a two-component model is less clear. The relationship between the SS(Fit) and the number of parameters is nearly linear. Again the Parafac models (PF2 and PF2-Orth) seem to be the best choice, and even though the orthogonal variant is marginally better, we decided to opt for the regular Parafac model. With respect to factorial invariance, the Parafac models incorporate invariant concept and scale spaces, and the correlations between scale components are constant over samples. The appropriateness of the Parafac models suggests that there is a considerable double-metric factorial invariance across the samples, only the size of the variances is different.

### 3.4. Results: non-invariant samples

For three-way models with double-metric invariance which are not necessarily invariant with respect to their links, we can compute the model fit for each sample. These fit measures can then be compared with the separate-analyses model to determine whether overall lack of interaction invariance is due to specific samples or whether differences are present between all samples.

#### 3.4.1. Differences in proportional fit of samples.

**Figure 3:**
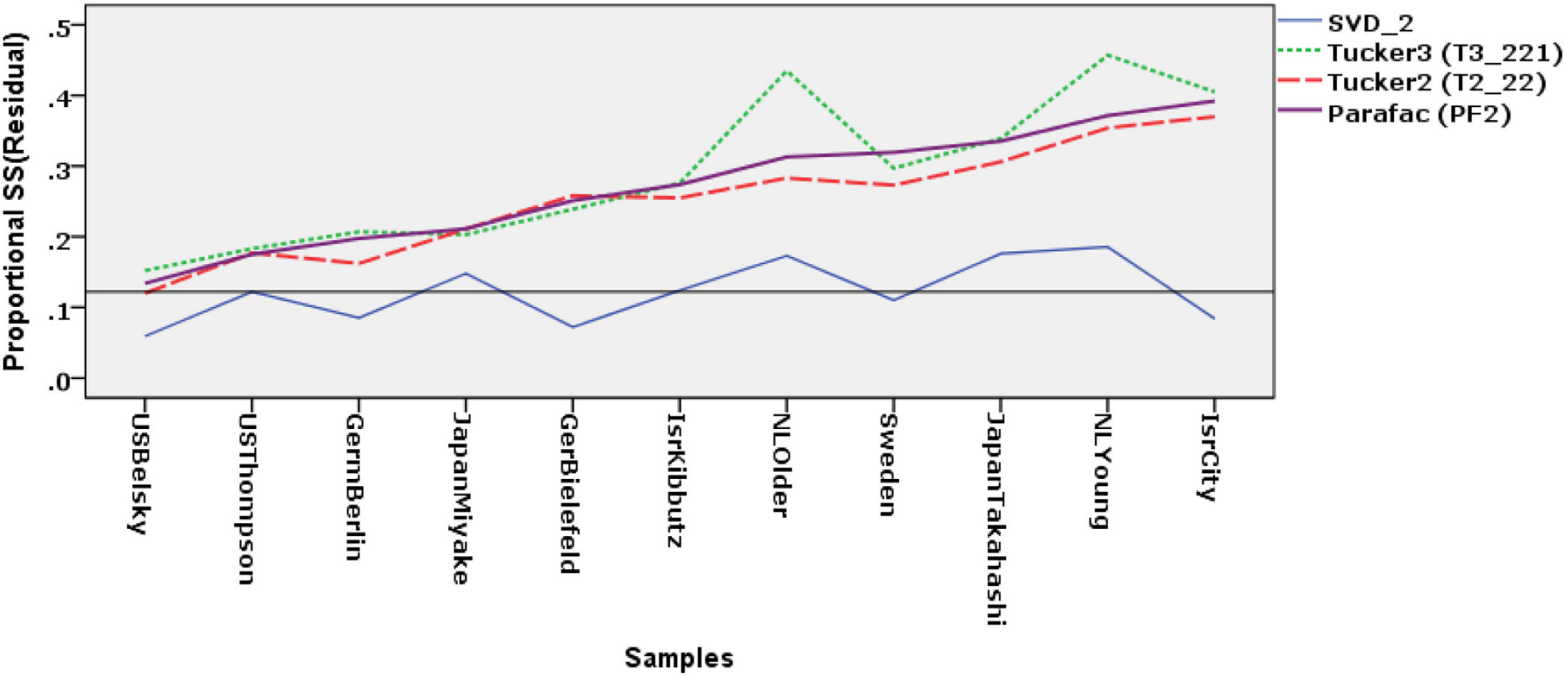
**Proportional Residual sums of squares per sample for four two-component models: Separate-analysis model (SVD-2), Parafac model (PF2), Tucker2 model (T2-22), and Tucker3 model (T3-221).** The samples are ordered on their fit based on the Parafac model with two components. Legend: US-Belsky (USBel), US-Thompson (USTho), Germany-Berlin (GerBe), Germany-Bielefeld (GerBi), Israel-Kibbutz (IsrKi), Israel-City (IsrCi), Japan-Miyake (JapMi), Japan-Takahashi (JapTa), Netherlands-Younger infants (NLYng), Netherlands-Older infants (NLOld) and Sweden (Swed).

For selected two-component models we calculated the proportional residual sums-of-squares PrSS(Res_*k*_) for each sample and connected these values per model in Figure 3. In the figure we have arranged the samples such that the lack of fit is increasing for the two-component Parafac model.

The solid line for the PrSS(Res) represent SVDs of the separate samples. We see that their PrSS(Res) fluctuate around the average value drawn as a horizontal line. In other words, a two-component SVD have about the same fitted sums of squares in all samples, but their concept spaces and their scale spaces are not necessarily equal.

In the case of strict model invariance all lines would be more or less horizontal because the lack of fit would be equal for all samples. This is not the case here. The relative difference in fit varies between the solutions for the separate samples and those of the models displayed in the figure. Thus, for the US samples on the left-hand side of the figure the metric invariant subspaces for the concepts and the scales are more alike to their own separate spaces than to the subspaces for the younger Dutch sample and Israel-City sample on the right-hand side. In particular, the PrSS(Res) for the two US samples is around 0.10 while it is around 0.30 for the younger Dutch and the Israel City sample.

All three metric invariant models displayed in Figure 3 show more or less the same pattern with an increasing loss of fit from left to right. Given that the models are more or less equivalent, we may choose to interpret the most restricted and thus most invariant model, i.e., the T3-221 or PF2 models. Figure 3 shows that the most right-hand samples fit marginally better, which is consistent with our earlier choice for this model. The Parafac model allows the components per sample to have a common oblique orientation with separate weights (*c*^*k*^_*ss*_) for the links between these common components. In this data set the younger Dutch sample and the Israel City sample need further investigation, because it is their configurations that are deviating most from the common pattern.

#### 3.4.2. Differences in strengths of links between concept and scale spaces across samples

**Figure 4:**
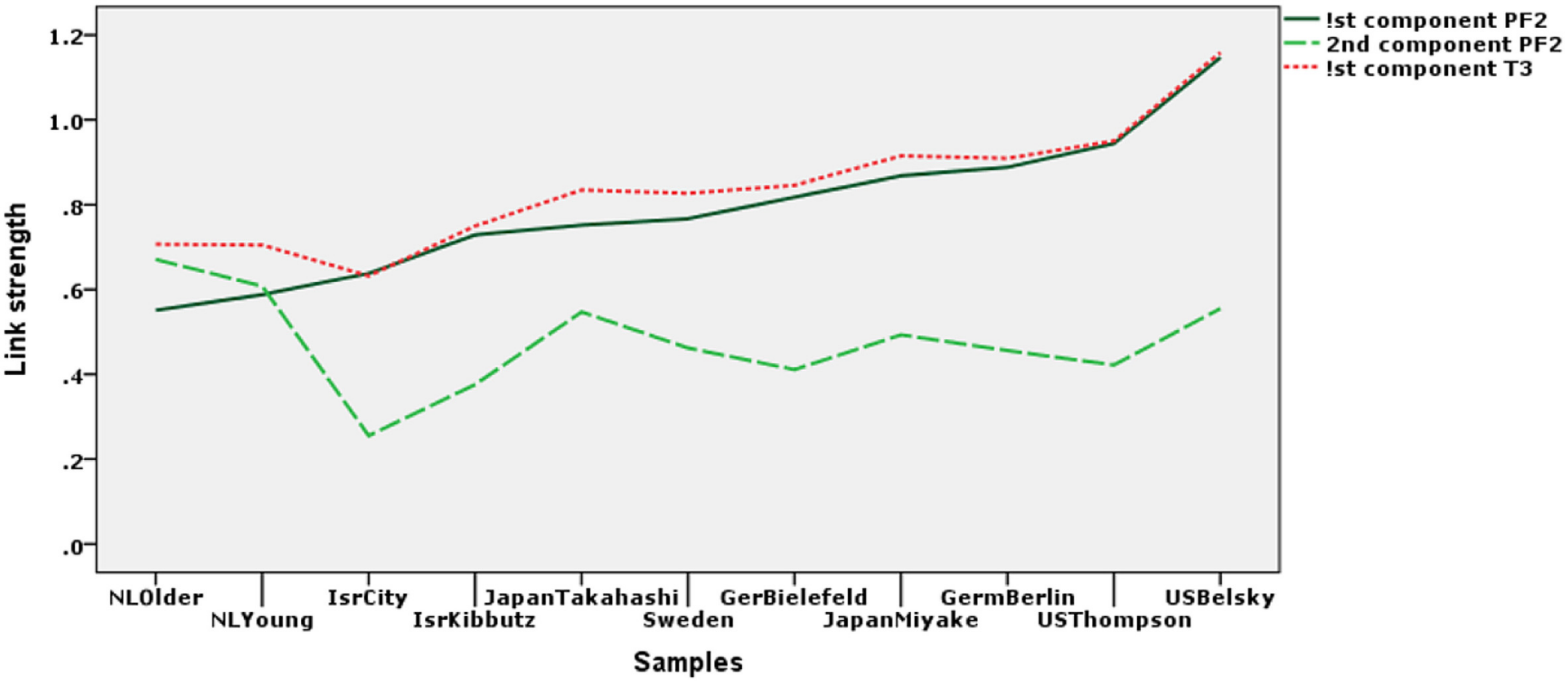
**The strength of the Parafac component links (*c*^*k*^_*ss*_) for each of the samples in principal coordinates.** The dotted line represents the weight or strength of the links from the T3_221_ model (*c*^*k*^) in principal coordinates. For the abbreviations of the samples names see Figure 3.

In Figure 4 we have plotted the link strengths *c*^*k*^_*ss*_ between the concept and scale components from the Parafac model with two components. The solid line represents the strengths of the links between the first components, *c*^*k*^_11_ and the dotted line the strength of the links for the second components, *c*^*k*^_22_. To provide a proper comparison these parameters have been depicted in principal coordinates. The third, dashed, line represents the weight parameter for each group according to the T3-221 model, *c*_*k*_; also in principal coordinates. The samples have been ordered so that the values for the first components, *c*^*k*^_11_, are increasing monotonically. The figure shows that *c*^*k*^_11_ and the *c*^*k*^ are almost equal, but that there is a small compensation of the *c*^*k*^ for the absence of the links for the second components *c*^*k*^_22_. Thus, the choice between the models should take into account whether the fluctuations of the *c*^*k*^_22_ are interpretable. At the same time the differences in the *c*^*k*^_22_ point to where we should look for lack of invariance.

If we want to find out what exactly are the differences between the samples, we have to explicitly compare the invariant concept and scale spaces with the separate sample spaces. Thus, this analysis could be extended to find the causes of the differences by examining the Tucker1 model for scales (T1B), and possibly the Tucker1 model for concepts (T1A), to assess whether it is the scale space or the concept space which is not invariant. We will not pursue this here. The procedure described above should primarily be seen as a proof of concept, rather than a detailed analysis of a particular case (see, however, the Appendix for a more substantive interpretation).

## 4. Results: an additional approach toward assessing invariance

In a paper comparing Japanese and Australian children in the way they show respect to adults, Kroonenberg and Kashima (1997) tackled assessing invariance in a different way, even if they did not explicitly refer to factorial invariance. The children were given a questionnaire in which they had to indicate both to what extent they did *show* a number of respectful behaviors (greet, help, stick up for, etc.) toward a number of adults (father, mother, teacher, etc.), and to what extent they felt they *should* do so. This resulted in a 5 (adults) × 7 (behaviors) × 4 (groups; Australian do, Australian should, Japanese do, Japanese should) three-way data set. Apart from a complete three-way analysis, the invariance was also assessed by first carrying out separate analyses for each of the four groups, and then using the adult space and/or the behavior space of one group as a restriction for the solution of another group. Essentially, of course, this is a cross-validating procedure, checking to what extent the parameter estimates in one group can also explain the variability in another group, or to what extent the two groups had invariant subspaces. However, one may equally see this as a procedure for establishing invariance. This procedure was referred to as *external analysis* by Van der Kloot and Kroonenberg (1985), because externally determined values for the parameters were used in fitting a particular data set.

For the Strange Situation data, this procedure could be used to investigate to what extent the separate solution of a sample is similar to that of another sample. In particular, the nature of the difference of the Dutch sample with respect to the other samples could be a focus of further analysis.

## 5. Conclusion

In this paper we have presented an approach toward assessing factorial invariance in two-way rating designs such as stimulus-response and semantic differential designs. Such designs generate fully-crossed three-way data which can be analyzed by three-way component models. True three-way models like the Parafac and Tucker models and their variants already incorporate various aspects of factorial invariance, in particular the double-metric invariance of the concept and scale spaces. The models vary in how they treat the relationships or links between the components. A hierarchy of models with increasing factorial invariance is outlined, running from no invariance for separate SVDs for each group, via single metric invariance for Tucker1 models, double-metric invariance for Tucker2 models, double-metric invariance and correlational invariance of Parafac models, to strict invariance for a very restricted Tucker3 model.

These models, and hence the nature of the invariance, can be assessed and compared via deviance plots showing the sum of squares of fit against the degrees of freedom. By connecting the relevant models by convex hulls in the plot, a comparative evaluation can be made and an appropriate model can be selected. Moreover, information supplied by the three-way analysis can be used to assess which group is more deviant from the invariant solution, and what the nature of such differences are.

The descriptive approach toward model selection, rather than using a formal testing paradigm, has been shown to work well for the example presented here. Data from a multinational collection of Strange Situation sessions (Sagi and Lewkowicz, 1987) were analyzed to demonstrate the effectiveness and usefulness of the model hierarchy for two-way rating data.

By investigating data from two-way rating designs we have extended the concept of factorial invariances beyond its standard definition. The future will have to show to what extent this extension is going to make an impact on the research on factorial invariance. For the present it seems that using the conceptualization presented here and the proposed hierarchy of three-way models, can shed light on differences and similarities between the invariance in two-way rating designs.

### Conflict of interest statement

The author declares that the research was conducted in the absence of any commercial or financial relationships that could be construed as a potential conflict of interest.

## Figures and Tables

**Figure A1:**
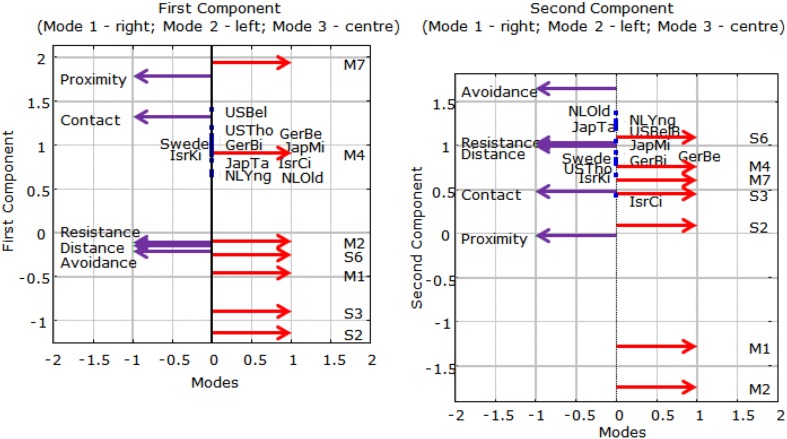
**Two-component Parafac analysis: Invariant components plus link strength of samples.** M*i* = Mother episode *i*; S*i* = Stranger episode *i*. For sample abbreviations, see Figure 3.

## Appendix

This appendix is presented here to offer some idea of the substantive outcomes of the invariance analysis of the Strange Situation.

For the Parafac model with two components, Figure A1 shows the normalized components of the three modes in two panels, both for the first and the second component as well as the strength of the links between them.

If there was complete strict invariance, the samples would have been superimposed in both panels at the value(*c**d*_*ss*_ (the T3-221Fixed model). If there would have been weighted strict invariance, the rank order and spacing of the samples for each of the components would have been equal i.e., at the values *c*^*k*^*d*_*ss*_ (the T3-221 model). As the figure shows, neither of these options was realized in the present data set, so that we must conclude that a double-metric invariant model (PF2) is the most restricted or invariant model that can be obtained.

The variances (or link strengths) of the components are *d*_11_ = 11.5 and *d*_22_ = 6.4, respectively (see Equation 5), so that the ratio of their importance in reconstructing the model is 1.8. Thus, the differences between the samples with respect to link strengths of the first components are about twice as large as those for the second components.

### A.1. First component

The left-hand panel of Figure A1 shows that securely attached (B) children show increasing Proximity seeking and Contact maintaining during the Mother episodes of the Strange Situation, as is evident from the increasingly higher coefficients on the first component. Seeking closeness to the mother is indicative of increasing stress during the procedure, which the B children try to alleviate by showing more and more proximity to the mother, i.e., showing a stronger secure attachment behavior. Treating the stranger with suspicion by staying at a distance is evident in the Stranger episodes; the coefficients remain negative but less so in S6 than in S3. Children's suspicion is decreasing slightly during the procedure but it is never absent. The other three scales all hover around zero, indicating that these behaviors of the children are not related to the two behaviors mentioned first. The US securely-attached children show the described patterns to the largest extent and the Dutch children the least. It is interesting to see that samples from the same country are generally close together with the largest difference between the two Israeli samples.

### A.2. Second component

The second components in the right-hand panel of Figure A1 describe mainly the avoidance, resistance and distance interaction behaviors toward the stranger, or stranger wariness. Such behavior is not typically present in the first two episodes but is present to a limited extent in the other episodes except for Episode 6, when it is the Stranger who returns rather than the Mother after the child has been alone in the fifth episode. With respect to the mother the situation is more complicated. There is a clear contrast between the earlier and later episodes, in that negative behavior toward the mother is not present in the beginning, but the children show a certain reserve when mother and child are reunited after the child has been alone with the stranger (Episodes M4 and M7).

These patterns are strongest in the Dutch and Japanese samples, as well as Belsky's US sample. Again, samples from the same country are generally close together except for the US samples.

### A.3. Invariance conclusion with respect to the B-children

The original research question was whether the structure behind the data from the two-way rating design for the secure B-children was the same across samples. The double-metric invariance embodied in the well-fitting two-component Parafac model indicates that this is indeed the case. However, the samples show a lack of invariance with respect to importance of the linkage between the components of the episodes and the scales. This difference is primarily a matter of relative importance of the two components. The first component embodies the increasingly secure attachment behaviors (Proximity seeking and Contact maintaining) over episodes, which is stronger in the US samples, especially in contrast with the Dutch samples. The second component represents ‘stranger wariness’ (Avoidance, Resistance and Distance interaction) which is especially strong in Episode 6, when the stranger rather than the mother returns, after the child as been alone in the fifth episode. To a lesser degree it also represents reservation toward the mother in the reunion episodes. These patterns are especially strong in the Dutch and Japanese samples and Belsky's US one.
